# Anal fistula with concomitant perianal cyst: A case report and literature review

**DOI:** 10.1097/MD.0000000000043887

**Published:** 2025-08-15

**Authors:** Zhihan Jiang, Kangze Wu

**Affiliations:** a Department of Colorectal & Anal Surgery, Shaoxing Yuecheng People’s Hospital, Shaoxing, PR China; b Department of General Surgery, Shaoxing People’s Hospital, Shaoxing, PR China; c The First Affiliated Hospital, Shaoxing University, Shaoxing, PR China.

**Keywords:** anal fistula, perianal cyst, recurrent infection, surgical management

## Abstract

**Rationale:**

Recurrent anal fistula remains a clinical challenge, especially when standard treatment fails to prevent relapse. Adjacent cystic infections may represent an underrecognized contributor to persistent or recurrent disease.

**Patient concerns:**

A patient with a long-standing history of perianal discomfort and multiple anal fistula recurrences presented with ongoing discharge and pain despite previous surgical interventions.

**Diagnoses:**

Clinical examination and intraoperative findings revealed a 2 × 2 cm infected cyst located adjacent to the recurrent anal fistula tract.

**Interventions:**

The patient underwent complete surgical excision of both the anal fistula and the adjacent infected cyst.

**Outcomes:**

Postoperative recovery was uneventful. The patient achieved sustained remission with no signs of recurrence observed during a 3-month follow-up period.

**Lessons:**

This case highlights a notable association between recurrent anal fistula and adjacent cystic infection. Cystic infection may serve as a hidden trigger for fistula reactivation and should be considered in patients with treatment-resistant perianal disease.

## 1. Introduction

Anal fistula, a chronic manifestation of acute anorectal pathology,^[1]^ typically presents as a nonhealing abscess post-drainage or persistent purulent discharge in the perianal or gluteal regions. Patients may experience intermittent rectal pain exacerbated by defecation, prolonged sitting, or physical activity, accompanied by malodorous discharge and pruritus.^[2]^ Current evidence attributes > 90% of anal fistulas to infected anal crypt glands,^[3–5]^ with Crohn disease,^[6]^ rectal foreign bodies,^[7–9]^ and infectious conditions (e.g., human immunodeficiency virus/acquired immune deficiency syndrome)^[10,11]^ being established etiological factors. We present a rare case of recurrent anal fistula associated with an infected perianal cyst.

## 2. Case report

A 69-year-old male presented with a 3-decade history of intermittent perianal purulent discharge and recent hemorrhagic exacerbation over 10 days. Despite previous fistulotomy, the patient reported persistent anal discomfort temporarily alleviated by spontaneous pus drainage. No history of perianal abscess or communicable diseases was documented. Physical examination demonstrated irregular anal contour with thrombosed hemorrhoids at 3, 5, and 9 o’clock positions (lithotomy position). A fistulous opening at 7 o’clock position, 3 cm from the anal verge, was connected to an indurated subcutaneous tract. Digital rectal examination revealed no palpable masses or blood-stained mucosa.

**Figure 1.**
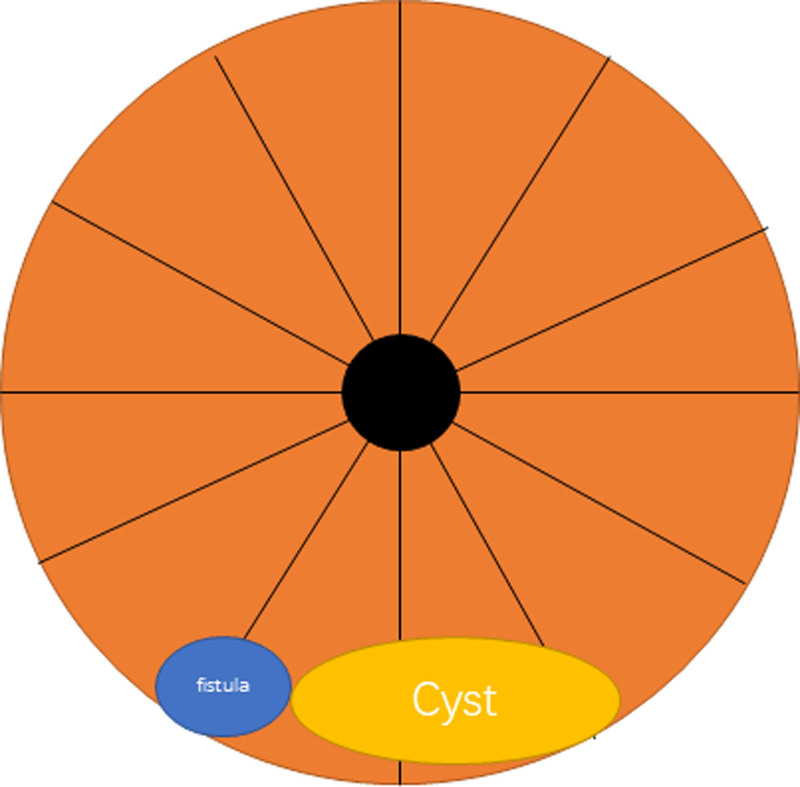
This schematic diagram depicts an orange circular cross-section representing perianal anatomy in lithotomy position, with radial divisions indicating anatomical quadrants (clock-face orientation). The central black circle denotes the anal lumen. The blue oval labeled “fistula” at the 7 o’clock position (left lower quadrant) and the yellow oval labeled “Cyst” at the 5 o’clock position (right lower quadrant) illustrate the spatial relationship between the pathological structures as documented intraoperatively.

**Figure 2.**
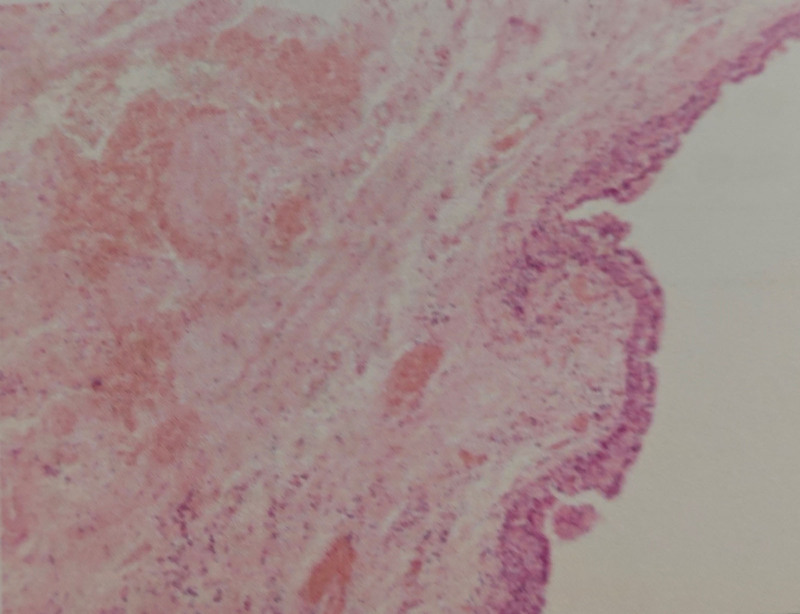
Histopathological findings of the specimen.

Subsequently, under spinal anesthesia, we performed fistulectomy and cyst excision. Intraoperative exploration revealed a 2 × 2 cm cystic lesion located at the 5 o’clock position, discharging turbid, xanthochromic fluid, and anatomically adjacent to the main fistula tract situated at the 7 o’clock position (Fig. 1). En bloc resection of the cyst along with complete fistulotomy was carried out, resulting in anatomical resolution. Postoperative histopathology showed that the fistula tract exhibited suppurative inflammation with fibrous tissue hyperplasia, while the cyst was identified as an epidermoid inclusion cyst with inflammatory cell infiltration (Fig. 2). Perioperatively, the patient received intravenous cefuroxime for infection prophylaxis. The surgical wound was packed with povidone–iodine gauze for drainage and was dressed daily. The patient was hospitalized for 6 days and was discharged home with instructions to continue daily dressing changes. Postoperative recovery was uneventful, and no signs of recurrence were observed during the 3-month follow-up period. Physical examination revealed no evidence of fistula recurrence.

## 3. Discussion

The clinical management of anal fistula continues to pose therapeutic challenges, particularly in cases with atypical presentations. Current epidemiological data indicate peak incidence among males aged 20 to 60 years (mean 40 years).^[12,13]^ While surgical excision remains the gold standard for achieving both infection control and sphincter preservation,^[14,15]^ our case underscores the critical importance of identifying occult infection sources. The patient’s postoperative recurrence despite adequate fistulotomy suggests residual cystic infection may have acted as a microbiological reservoir, a mechanism not previously described in fistula pathogenesis (Table 1).

**Table 1:** Comparative analysis of clinical characteristics: present case versus literature-reported cases of anal fistula with perianal cyst.

	Current case	Typical cases in literature
Age/gender	68-years-old, male	Mean age 40 years (range 20–60), male predominance
Medical history	- 30-year intermittent purulent discharge - Previous fistulotomy history - Denied perianal abscess history	- Chronic manifestation of acute anorectal pathology - Potential Crohn disease/rectal foreign body association
Clinical manifestations	- Perianal purulent discharge - External opening at 7 o’clock - No masses on digital examination	- Defecation-related pain - Perianal discharge/pruritus
Intraoperative findings	- 2 × 2 cm cyst at 5 o’clock position - Fistula tract at 7 o’clock - Anatomical proximity between cyst and fistula	90% cases show cryptoglandular infection origin
Surgical intervention	Cyst excision + fistulotomy	Primary methods: fistulotomy/seton placement
Unique pathological factor	Coexisting perianal cyst infection	HIV infection, rectal foreign bodies

Notably, conventional preoperative assessments including digital examination and basic laboratory investigations failed to detect the cystic lesion, highlighting the diagnostic limitations in complex perianal pathologies. This observation aligns with existing evidence demonstrating 69% to 81% healing rates depending on fistula complexity,^[16,17]^ emphasizing the prognostic value of complete infection source control. From a pathophysiological perspective, the cyst’s anatomical proximity to the fistula tract supports potential cross-contamination through local tissue planes, though the exact mechanism warrants further investigation (Table 1).

## 4. Conclusion

This first documented case of anal fistula associated with infected perianal cyst expands our understanding of recurrence mechanisms in anal fistula. The findings emphasize the necessity of meticulous intraoperative exploration and suggest considering cystic lesions in the differential diagnosis of refractory fistula cases. Future studies should investigate the prevalence of such associations.

## Author contributions

**Conceptualization:** Zhihan Jiang, Kangze Wu.

**Data curation:** Zhihan Jiang, Kangze Wu.

**Writing – original draft:** Zhihan Jiang.

**Writing – review & editing:** Kangze Wu.
